# Feasibility of substituting old corrugated carton pulp with thermal alkali and enzyme pretreated semichemical mechanical rice straw pulp

**DOI:** 10.1038/s41598-022-07482-z

**Published:** 2022-03-03

**Authors:** Yu-Hsun Lai, Hao-Chen Sun, Ming-Hui Chang, Ching-Chin Li, Jiann-Gwo Shyu, Yuan-Shing Perng

**Affiliations:** 1 Department of Forestry, National Chung Hsing University, Taichung, 40227 Taiwan; 2 Agricultural Chemistry Division, Taiwan Agricultural Research Institute, Taichung, 413008 Taiwan; 3 Wood Cellulose Division, Taiwan Forestry Research Institute, Taipei, 100051 Taiwan

**Keywords:** Environmental sciences, Materials science

## Abstract

In this study, we separately used a laboratory Hollander beater, a pilot scale 12″ single-disc refiner and an expanded trial with a commercial paper mold mill to investigate the feasibility of using thermal-alkali/enzyme pretreated rice straw semi-chemical mechanical pulp to substitute portions of old corrugated carton board (OCC) pulp in the paper industry. In the laboratory plan, sequential treatments of NaOH at a 5–10% dosage and enzymes at a 0.2–4% dosage were applied to rice straw, followed by beating using a Hollander beater for 1–2 h to complete the rice straw semi-chemical mechanical pulping process. When the NaOH dosage, enzyme dosage and refining time were 10%, 0.2% and 1 h, the best quality rice straw pulp was obtained. Along with the increase in NaOH dosage, the pulp freeness decreased significantly, and the pulp accepted rate increased. Enzymatic treatment enhanced rice straw quality only after NaOH dosage treatment, which then reacted with rice straw to increase the quality of pulp. In the expanded trial, the rice straw semi-chemical mechanical pulp was blended with OCC pulp (0%, 25%, 50%, 75% and 100%) to form handsheets. Along with an increase in rice straw proportions, the tensile index, burst index, and ring-crush index increased by 109–200%, 13–196%, and 124–187%, respectively. In an online commercial paper mold mill trial, blending rice straw pulp with OCC could successfully make paper-mold egg cartons, with both mill operation and product smoothness appearance being highly acceptable.

## Introduction

In recent years, along with the development of the pulp and paper industry, the demand for papermaking raw materials has also increased. This leads to gradual depletion of woody resources and escalating prices. There were 3 genres of papermaking raw materials: wood, nonwood, and recycled wastepaper. At present, globally 92% of all material comes from wood fibers, coniferous or hardwood trees^1,2^; nonwood fiber accounts for less than 8% of all raw materials. Of these, China consumes the most (70.7%), followed by India (8.0%), Pakistan and other regions (2%)^2–4^. With advances in environmental consciousness and wastepaper recycling technology, by 2003, the global use of secondary fiber had exceeded virgin fiber. In 2016, the global fiber consumption was 425 million tonnes; of these, secondary fiber accounted for 57% and virgin fiber accounted for 43%.

Driven by environmental protection policies, in recent years, various paper mills have begun to use large amounts of wastepaper. However, in 2019, China implemented a policy of prohibiting waste importation and to transpire with zero wastepaper imports by 2021. This led to serious shortfalls of papermaking raw materials. Seeking other papermaking materials is one of the solutions to the problem. How to effectively use nonwood fiber, such as agroforestry residues and perennial plants, etc. to compensate for the shortfalls in papermaking materials has become a stressed R&D item^2^.

Rice straw is a nonwood fiber material; it is one of most abundant agricultural wastes and a renewable biomass resource. In China, each year, a total of 180 million tonnes of rice straw is produced, which ranks 3rd among agricultural wastes, and it is surpassed only by whether straw and corn stalks are produced^5,6^. In Taiwan, each year, 1.80 million tonnes of rice straw is produced^7^. In Asia or some developing countries, a portion of rice straw is shredded and shortened for manure and fodder, and most of the rest is burnt, which not only wastes resources but also creates serious air pollution, often causing health hazards. If thick smog diffuses to the surrounding roadway, it also causes traffic safety problems^8,9^.

Traditional pulping methods are mainly divided into chemical, mechanical and combined methods^10,11^. Rice straw mainly uses the traditional soda method to pulp, and typical operational conditions entail a NaOH dosage of 12–16% and a temperature of 140 to 170℃. The fiber characteristics and chemical compositions of rice straw, wheat straw and bagasse are shown in Table 1. From the table, we can perceive that when rice and wheat straw are compared to bagasse, the fiber length and width are smaller; lignin content is lower, cellulose and hemicellulose content is also lower, whereas ash and silica content is higher^12^. However, the high ash content, particularly silica, will lead to problems in the recycling of cooking liquor, which will often lead to environmental pollution and become a major hindrance in commercialization^1–4,13,14^.

**Table 1:** Characteristics of rice straw, wheat straw and bagasse^12^.

Materials	Fiber length (mm)	Fiber wide (μm)	Cellulose (%)	Hemicellulose (%)	Lignin (%)	Ash (%)	Silica (%)
Rice straw	1.41	8	28–36	23–28	12–16	15–20	9–14
Wheat straw	1.48	13	29–35	26–32	16–21	4–9	3–7
Bagasse	1.70	20	32–44	27–32	19–24	1.5–5	0.7–3

In woody fiber materials, the encrustation of lignin limits the accessibility of cellulosic cores. Only through suitable pretreatments is cellulose extracted easier, which allows subsequent pulping operation^15^. The methods of pretreatments generally include chemical, physical and biological means. Chemical pretreatments are more traditional and can generally deal with lignocellulosic substrates. However, because chemical agents are required, wastewater pollution often entails. For instance, alkali pretreatment is akin to kraft pulping, and the removed hemicellulose and lignin can further degrade the crystallinity of cellulose and the degree of polymerization of hemicellulose and lignin; in the meantime, the porosity of cellulose and its reactivity and accessibility are also increased^16,17^. Through NaOH pretreatment, it can degrade phenol groups and remove lignin and pectin, so that the functional groups of macromolecules can be partially dissolved^18^. Physical pretreatment generally requires a shorter time, but due to the high energy consumption and destructive force, it easily causes damage to fiber^19,20^. Biological pretreatments have milder reaction conditions, which reduces the environmental damage it causes to a large extent; however, the efficiency is relatively low^21^.

In this study, we separately applied a laboratory Hollander beater, an expanded pilot scale 12″ single-disc refiner, and commercial paper mold mill online testing to investigate the feasibility of using thermal alkali/enzymatic pretreatments to rice straw semi-chemical mechanical pulp to substitute the pulp for old corrugated carton (OCC) pulp. Then, the rice straw semi-chemical mechanical pulp was blended with OCC pulp to form handsheets, and the tensile, burst and ring crush strengths were measured. In the commercial paper mold mill operation, the rice straw semi-chemical mechanical pulp was blended with OCC pulp and formed into paper egg carton to verify that the pulp is applicable to the paper mold and papermaking industry.

## Experimental

### Materials

The rice straw obtained in this study was provided by the Taiwan Agricultural Research Institute, which was cut into pieces with a length of ca. 10–15 mm, moisture content of approximately 10%, then stored in a gunny bag. Storage time is 2 years. The collection of rice straw was in compliance with relevant institutional, national, and international guidelines and legislation. The OCC pulp was provided by the Erlin mill of Long-Chen Paper & Packaging Ltd. The OCC pulp’s average fiber length is 1.39 mm and freeness 613 mL. The OCCs were disintegrated, washed and dewatered and then stored in a refrigerator. The formulated enzyme was provided by RisingStar Biotech Ltd. of Kwangchou, China, designated product no. 9888, with the best bioactivity at pH 5.5. The NaOH and HCl used were reagent grade and provided by Shangmei Instruments, Taipei, Taiwan.

### Method

This study was proceeded in three stages. In the first stage, rice straw semi-chemical mechanical pulp was prepared. Through the use of NaOH and enzymes, sequential pretreatments were conducted, and then a Hollander beater was used to refine the pulp. The experimental variables were NaOH dosage (5–10%), enzyme dosage (0.2–4%), and refining time (1–2 h). The experimental design used a 2^3^ factorial design to examine the main effects and interactions. The factorial of variables are described in Table 2. The central point was replicated 3 times to allow calculation of the system standard deviation. The total number of experiments were 11 sets. The parameters in Table 2 were referred to Lazorenko et al. (2020)^22^, enzyme supplier’s recommended dosage, and the preparative experiments.

**Table 2:** The 2^3^ factorial experimental design.

Parameters	− 1	0	+ 1
A: NaOH dosage (%)	5	7.5	10
B: Enzyme dosage (%)	0.2	2.1	4
C: Refining time (h)	1	1.5	2

**Figure 1:**
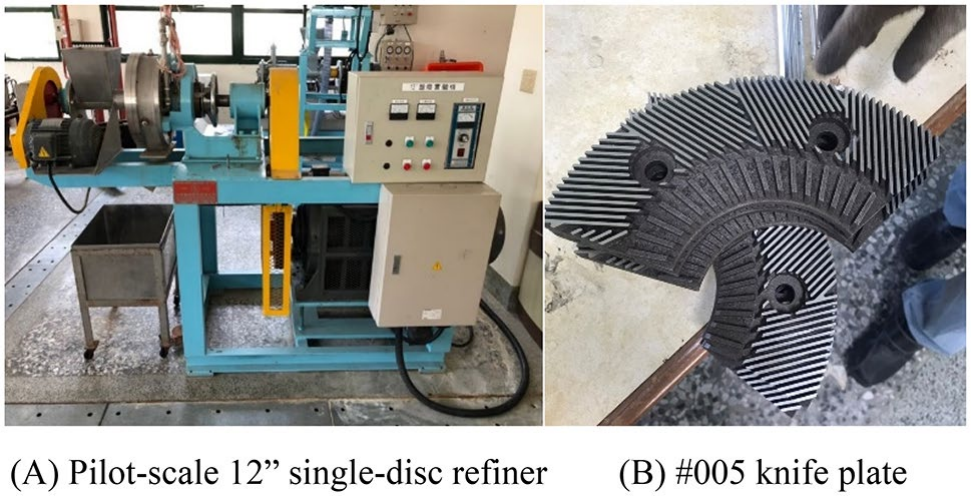
Photos of the pilot-scale 12″ single-disc refiner (**A**) and the #005 knife plate (**B**).

**Table 3:** The factorial experimental design results of the pulp properties.

Group	NaOH dosage (%)	Enzyme dosage (%)	Refining time (h)	Freeness, before refining (CSF mL)	Freeness, after refining (CSF mL)	Accepted fiber rate (%)
1	5	0.2	1	764	588	32.3
2	10	0.2	1	552	220	44.7
3	5	4	1	804	517	31.3
4	10	4	1	402	163	44.4
5	5	0.2	2	756	602	31.7
6	10	0.2	2	488	109	53.0
7	5	4	2	804	341	31.6
8	10	4	2	439	85	52.5
9–1	7.5	2.1	1.5	666	156	32.4
9–2				669	153	31.7
9–3				666	187	32.7
Group 9’s mean				667	165	32.3
Group 9’s standard deviation				1.7	18.8	0.5

**Figure 2:**
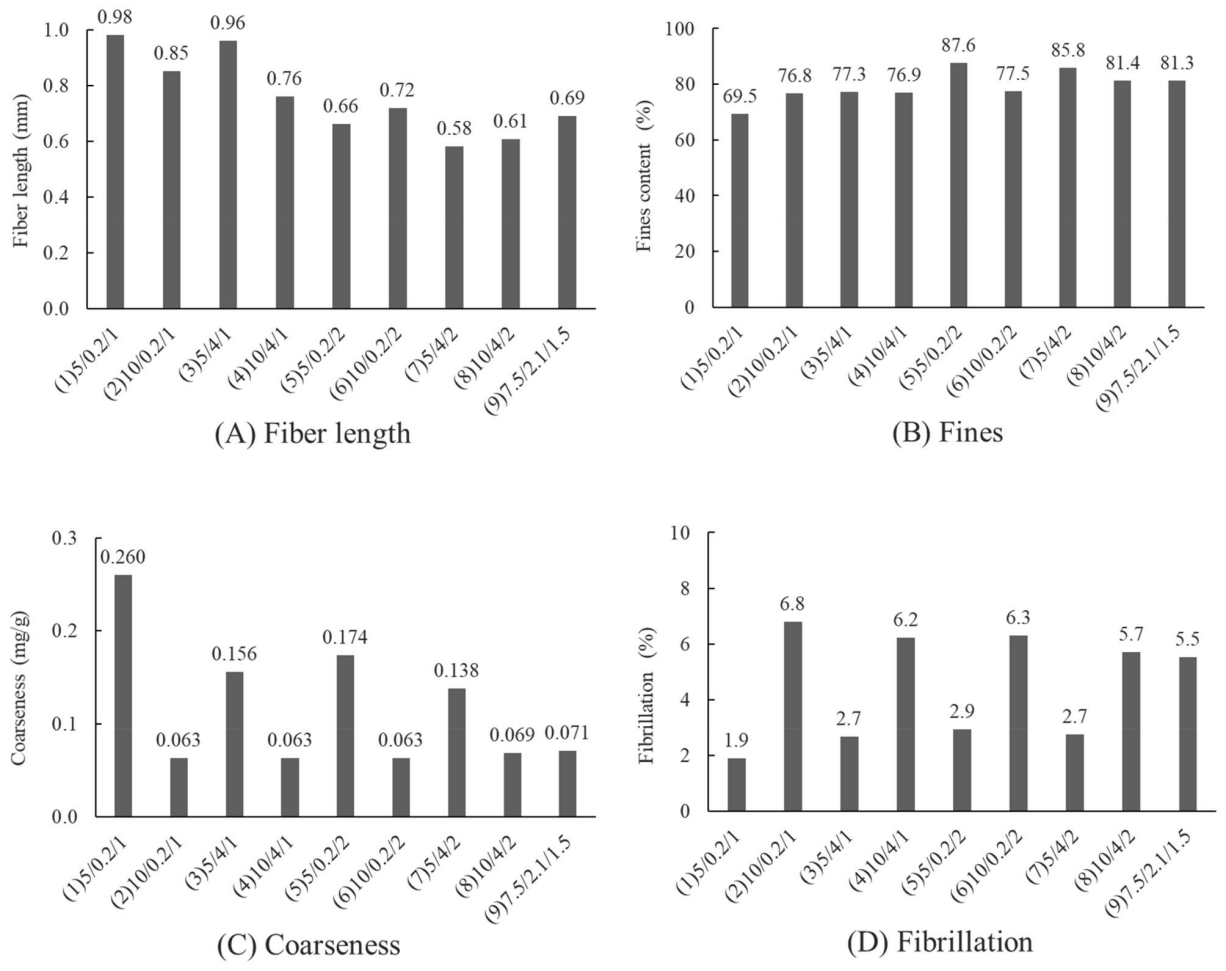
Effects of different treatment groups of rice straw semi-chemical mechanical pulp on fiber length (**A**), fines (**B**), coarseness (**C**) and degree of fibrillation (**D**).

**Figure 3:**
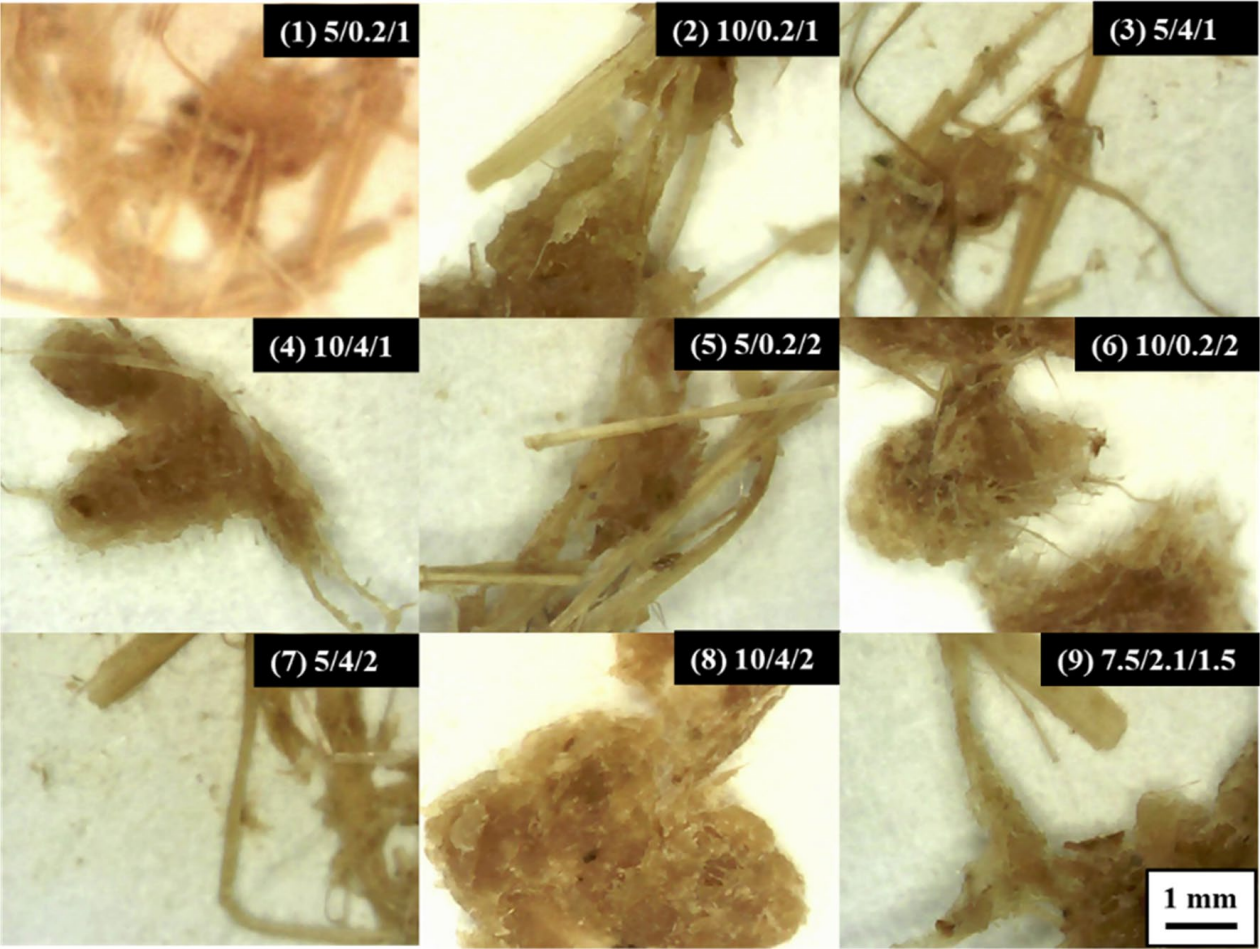
Whole spectral micrographs of rice straw semi-chemical mechanical pulp at a magnification of 50x. Note: The graph set number represents NaOH dosage (%), enzyme dosage (%) and refining time (h).

**Figure 4:**
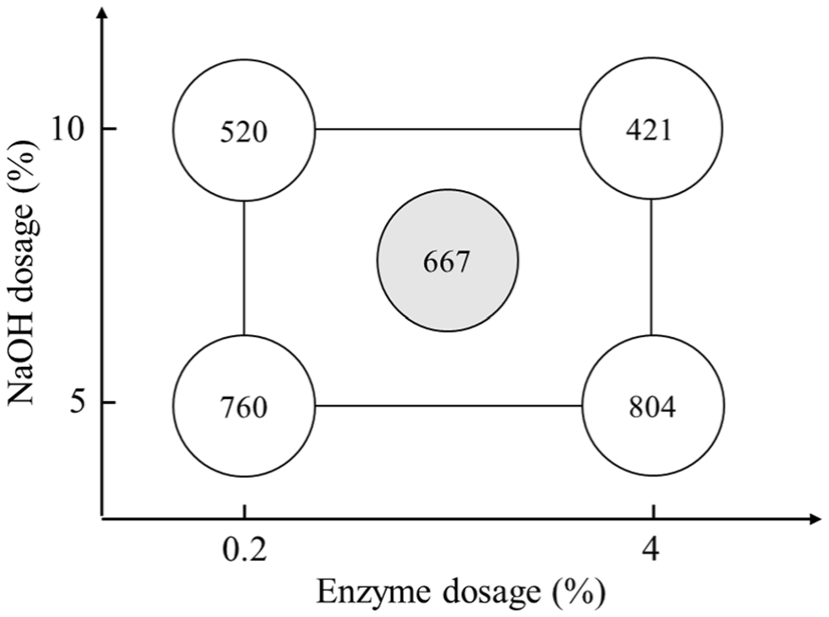
Factorial analysis of rice straw semi-chemical mechanical pulp freeness (CSF, mL) before refining.

**Figure 5:**
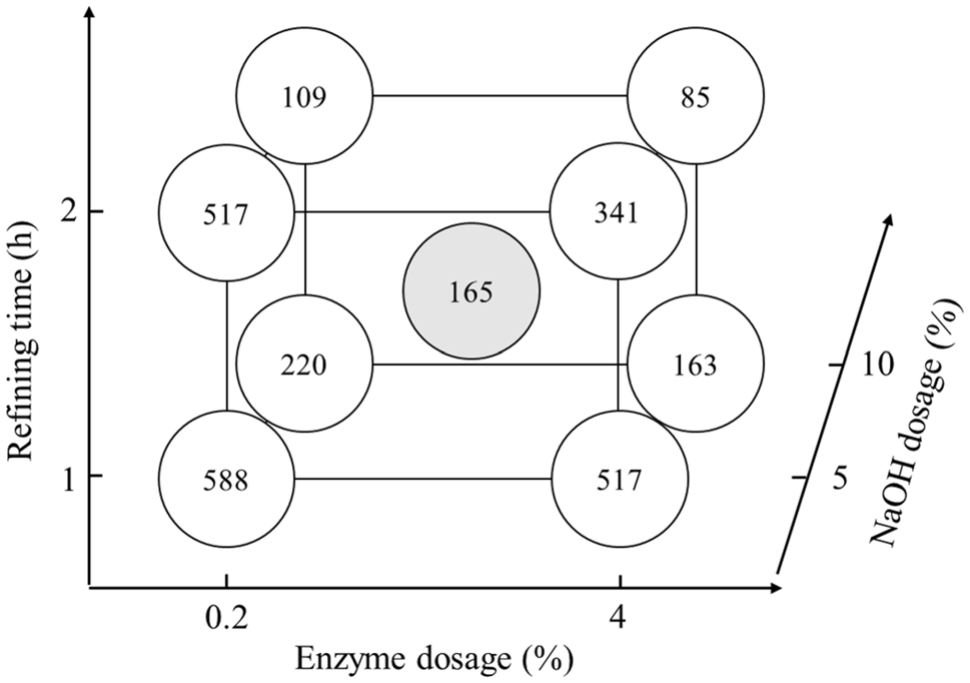
Factorial analysis diagram of rice straw semi-chemical mechanical pulp with respect to postrefining pulp freeness (CFS, mL).

**Table 4:** The effect values of 2^3^ factorial designs with respect to postrefining pulp freeness variables.

Factor	The assessed value	Significance
Average =	453.3 ± 18.0	–
Factor A =	−227.1 ± 18.0	Yes
Factor B =	−6.4 ± 18.0	–
Factor C =	21.9 ± 18.0	Yes
AB interaction =	62.1 ± 18.0	Yes
AC interaction =	−7.4 ± 18.0	–
BC interaction =	11.1 ± 18.0	–
ABC interaction =	−3.6 ± 18.0	–

The assessed value =  ± ts/√(N⁄4) = 4.5

A: NaOH dosage (%), B: Enzyme dosage (%), C: Refining time (h).

**Figure 6:**
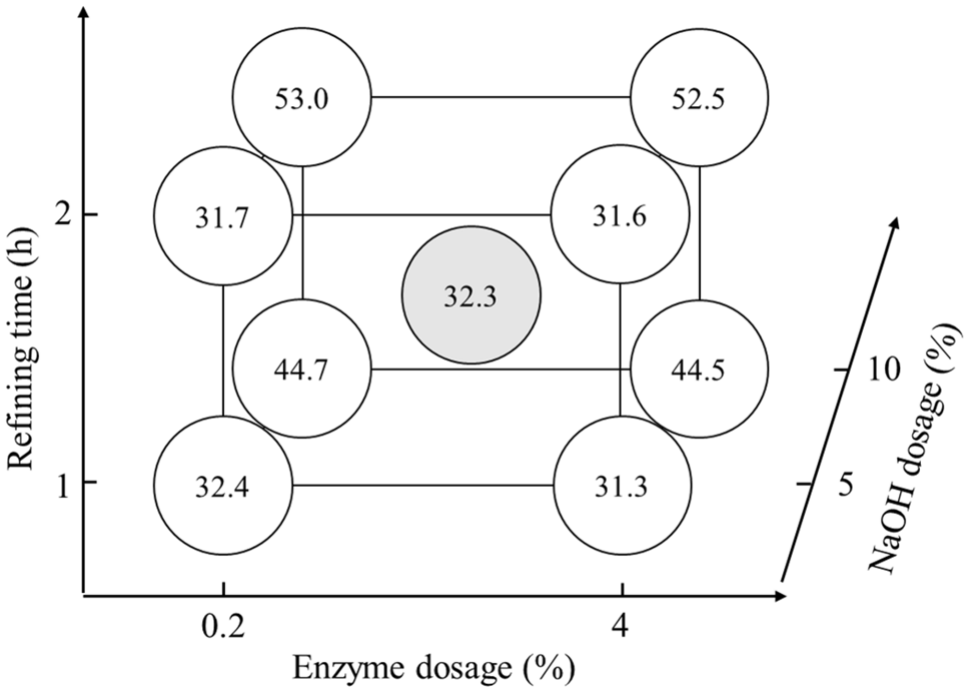
Factorial analysis of rice straw semi-chemical mechanical pulp with respect to acceptable fiber rates.

**Table 5:** Variable effects of the acceptable fiber rate in the 2^3^ factorial designs.

Factor	The assessed value	Significance
Average	42.8 ± 4.5	–
Factor A	11.6 ± 4.5	Yes
Factor B	−0.5 ± 4.5	–
Factor C	4.0 ± 4.5	–
AB interaction	−5.2 ± 4.5	Yes
AC interaction	4.2 ± 4.5	–
BC interaction	0.2 ± 4.5	–
ABC interaction	0.3 ± 4.5	–

The assessed value =  ± ts/√(N⁄4) = 4.5

A: NaOH dosage (%), B: Enzyme dosage (%), C: Refining time (h).

**Table 6:** The factorial experimental results of handsheet properties.

Group	Blending ratio (OCC/Rice straw)	NaOH dosage (%)	Enzyme dosage (%)	Refining time (h)	Tensile index (Nm/g)	Burst index (kPa*m^2^/g)	Ring crush index (kgf*m^2^/g)
1	50%/50%	5	0.2	1	15.27	0.71	5.12
2		10	0.2	1	29.61	1.42	7.37
3		5	4	1	16.46	0.73	6.10
4		10	4	1	27.00	1.20	7.90
5		5	0.2	2	18.05	0.82	5.95
6		10	0.2	2	31.54	1.49	7.02
7		5	4	2	28.08	1.25	11.94
8		10	4	2	28.26	1.30	6.79
9–1		7.5	2.1	1.5	25.98	1.33	8.28
9–2					28.73	1.26	7.93
9–3					27.58	1.27	8.13
10	100%/0%	–	–	–	18.86	0.92	5.17
Group 9’s mean					27.4	1.3	8.1
Group 9’s standard deviation					1.38	0.04	0.18

In accordance with Table 2, the rice straw was added at 80℃ and reaction consistency of 5.8% to the reaction vessel with NaOH dosage of 5%, 7.5%, and 10% separately and reacted for 2 h. After thorough washing, then at 60℃, pH 5.5 and consistency of 5.8%, the enzyme was added (0.2, 2.1 and 4%) and reacted for 2 h. After further washing, the prepared rice straw was beaten with a Hollander beater at a fixed weight of 1 kg and applied different refining time (1, 1.5, 2 h) to complete the preparation of rice straw semi-chemical mechanical pulp. Then, the pulp properties were analyzed. These included rice straw pulp fiber morphological features, accepted rate (including coarse fibers and fines) and pulp freeness. It was followed by handsheet preparation, which used a standard handsheet mold to form handsheets of different proportions of rice straw pulp with OCC (50 and 100%) to circular handsheets of 100 g/m^2^. The handsheet properties, such as tensile, burst, and ring crush strengths, were then measured. Finally, through pulp property analyses and handsheet physical properties, the best options for preparing the pulp were determined.

In the second expanding stage, the best rice straw pretreatment conditions were used to pretreat rice straw, then a pilot-scale 12″ single-disc refiner rotating at 1000 rmp, refining consistency of 5%, and 4 repeated passes to refine the pulp and produce the mechanical rice straw. The pulp was measured for the accepted rate and pulp freeness. Finally, the pulp was formed into handsheets with a standard handsheet mold using rice straw pulp and OCCs of different blend ratios (0%, 25%, 50%, 75% and 100%) to form 100 g/m^2^ circular handsheets. The physical properties of the handsheets thus obtained, including tensile, bursting and ring crush strengths, were measured.

The third stage was a commercial paper mold mill online test. We entrusted the Changtai Environmental Technology Ltd. to blend rice straw with OCC pulp and use the mill facilities to produce paper mold for storing eggs to evaluate the on-site operational performance and the feasibility of substituting OCC with blended rice straw pulp.

### Procedure

In the first stage of rice straw semi-chemical mechanical pulp preparation, rice straw was first thermally alkali pretreated. Add 3 L of water and NaOH to an electromagnetic heated water bath, control the temperature to 80 °C, and then 175 g o.d.. Activate the D.C. stirrer to a spinning rate of 600 rpm reacting for 2 h, then washing the pulp. Subsequently, taken 175 g o.d. of the thermal-alkali pretreated rice straw and 3 L of water in an electromagnetic heated water bath, using a pH meter and 1% HCl solution to adjust the suspension to the optimal bioactive environment (pH 5.5, 60℃). Then, based on the treatment dosage, enzyme was added and stirred with a glass rod to make the suspension uniform. Activate the D.C. stirrer to a spinning rate of 600 rpm and reacting for 2 h, then washing the pulp to complete the enzymatic pretreatment.

After completing thermal-alkali and enzymatic pretreatments, the pretreated rice straw was subjected to Hollander beater refining in accordance with TAPPI T 200 sp-15 using a Hollander beater made by Lesson Industrial CO., Ltd., New Taipei City, Taiwan. Oven-dry weight 175 g of pretreated rice straw was added to the Hollander beater, and 10 L of water was added. Under no weight load, start the motor and proceed with 30 min of pulp disintegration. Then, 1 kg weight was added to proceed with the refining operation to complete the preparation of rice straw semi-chemical mechanical pulp. After treatment, the rice straw semi-chemical mechanical pulp was dewater with a dewatering machine and then kept in a refrigerator to store and to provide subsequent pulp property analysis.

Ensuing the above, and in accordance with TAPPI T 205 sp-12 (2018), the rice straw chemical pulp was formed into handsheets using sheet mold made by Lesson Industrial CO., Ltd., New Taipei City, Taiwan. First, pulp preparation was performed to simulate the grammage of the corrugating medium by forming a handsheet of 100 g/m^2^. The handsheet samples were stored at 23.0 ± 1.0 °C temperature and 50.0 ± 2.0% relative humidity for at least 24 h in accordance with TAPPI T 402 sp-08, and then the handsheet was removed and the subsequent physical tests proceeded.

Based on the first-stage experimental results, the best pulping conditions were selected for the second-stage expanded experiments. A pilot-scale 12″ single-disc refiner (New Bonafide Co., New Taipei City, Taiwan) was used, and the knife plates used were #005 plates for refining (Feng Ji Industrial Co., Fengyuan, Taiwan) to replace the laboratory-scale Hollander beater to refine the pulp. The 12″ single-disc refiner and #005 knife plate are shown in photos of Fig. 1. For the operational condition selection, we aimed to reach the best refining time pulp freeness as the single-disc refiner option. Based on the preparative experiments, we found that the repass was limited to 4 times. After 4 cycles, the additional refining did not have a significant effect on the pulp freeness. In practice, we first subjected 3 kg of rice straw to the best pretreatment conditions, adjusted the volume consistency to 5%, activated the single-disc refiner, set the rotor plate to 750 rpm, and then injected pulp suspension to proceed with refining. After the rounds of refining, the pulp was dewatered with a dewater unit that completed the optimal conditions of disc-refiner pulp preparation. Afterward, the disc-refined rice straw pulp was blended according to the set ratios with OCC pulp to form handsheets. Of these, the rice straw pulp blending ratios were 0%, 25%, 50%, 75%, and 100%. Finally, the completed handsheets were physically tested for tensile, burst, and ring crush strengths.

The third stage was a commercial paper mold mill online test. Sixty kg of rice straw pulp was delivered to the cooperating Chang-Tai Co. Upon blending with the 100% OCC-based and 20% rice straw pulp, the raw materials after pulp disintegration were turned into pulp, and then vacuum was applied to the molding to form the products. After drying, hot press shaping was applied according to demand and to strengthen external shaping and structural strength to complete the paper molding production.

### Pulp properties

1. Fiber morphological characteristics
  A whole-spectral microscope and fiber morphological analyzer (Valmet FS5, Victoria, Australia) were used to analyze the fiber morphologies of the rice straw pulp, including fiber length (weight-weighted length), coarseness, degree of fibrillation, and fines amounts. Each set of samples was replicated 6 times. The whole-spectral microscope (500X series, Gaopin Precision Instrument Co. Ltd., Kun Shan, China) was linked to a computer that adjusted the magnification to 50 times to facilitate observation of the morphologies of rice straw pulp.
2. Pulp freeness
  Pulp freeness was measured according to TAPPI T 227 om-17 using a freeness instrument (Lesson Industrial CO., Ltd., model 316, Taipei, Taiwan). Each set of samples was measured for freeness in 3 replications.
3. Accepted fiber rate
  The accepted fiber rate was measured according to TAPPI UM 240 by using filter screens with mesh sizes of 10 and 150 mesh to separate fiber from shives in separated tests. The results of the screening are defined as follows: shives > 10 mesh; fiber < 10 mesh -150 mesh; and fines < 150 mesh. Each set of samples was replicated 3 times for the accepted rates.

### Handsheet properties

1. Tensile strength
  The tensile strength of the handsheets was tested according to TAPPI T404 using a constant rate elongation instrument (Lesson Industrial CO., Ltd., New Taipei City, Taiwan).
2. Burst strength
  Handsheets were tested according to TAPPI T 403 om-15 for burst strength. A Lesson Industrial CO., Ltd. burst tester (New Taipei City, Taiwan) was used for the test.
3. Ring crush strength
  The ring crush strength of the handsheets was tested according to TAPPI T818 (R2018) using a ring crush tester (Lesson Industrial CO., Ltd., New Taipei City, Taiwan) for the test.

## Results and discussion

### The first stage experiments: Laboratory experiments

#### Pulp properties

In the first stage, rice straw was subjected to thermal-alkali and enzymatic pretreatments and then beaten with a Hollander beater to prepare the rice straw semi-chemical mechanical pulp. The resulting pulp properties based on the factorial experimental design are shown in Table 3. The mass loss of rice straw after thermally alkali pretreatment is approximately 14.3–25.6%. The central point condition (group 9) with 3 replications gave pulp freeness standard deviation of 1.7 mL (before beating) and freeness standard deviation of 18.8 mL (post-beaten), standard deviation of acceptable fiber rate was 0.5%, and degree of freedom of 2.

1. Fiber morphologies
  The thermal alkali/enzymatic pretreated and beaten rice straw semi-chemical mechanical pulps were analyzed for their fiber morphologies. The effects of the rice straw semi-chemical mechanical pulps with respect to fiber length (A), fines content (B), coarseness (C) and degree of fibrillation (D) are illustrated in Fig. 2. According to Fig. 2(A) and (B), when the pulp beating time increased from 1 to 2 h, the fiber length decreased significantly. In addition, the fines proportion increased. Among the factors, the dosage of NaOH and enzyme effects on fiber length were relatively minor. According to Fig. 2(C) and (D), when NaOH increased from 5% to more than 7.5%, the coarseness of the pulp was significantly reduced, and the degree of fibrillation showed a significant increase. Among the set (2) 10/0.2/1, NaOH dosage was at maximum, whereas enzyme dosage and beating time were at a minimum, the coarseness and degree of fibrillation of the pulp had already achieved optimal. If we raised the enzyme dosage and beating time, excessive damage to the fiber could cause the handsheet properties to decrease instead.
  Figure 3 shows the whole spectral rice straw micrographs. The numbers of the groups represent NaOH dosage, enzyme dosage and beating time. According to sets (1), (3), (5), and (7) of the figure, when the NaOH dosage was 5%, coarser shives abound, and defibrillated fiber was less abundant. However, when set (2), (4), (6), (8), and (9), micrographs indicated that when NaOH dosages were raised to 7.5% or more, large, coarse shives showed significant reduction, defibrillation and fibrillation became more effective. Based on these results, when the NaOH dosage increased from 5% to 7.5% or more, rice straw softened, which enhanced the subsequent enzymatic reaction and refining efficacy, causing the coarse fiber proportion of the rice straw pulp to decrease, and the pulp became more desirable. Among these, along with the addition of enzyme dosages and refining time increase, the defibrillation of the rice straw pulp showed some optimization trend, which was particularly evident at conditions of high alkali dosages.
2. Pulp freeness
  After thermal alkali and enzymatic pretreatments, the before and after refining pulp freeness was measured. Figure 4 shows the rice straw semi-chemical mechanical pulp group performances before refining the pulp freeness values. Each variable is described in Table 2. Three replications were carried out for the midpoint conditions. Before refining, the average pulp freeness was 667 mL, and the standard deviation of the freeness was 11.6 mL. At lower NaOH dosages, along with increasing enzyme dosages, the freeness before refining increased from 760 to 804 mL, showing an increasing trend. According to Singh and Bhardwaj (2011), this was probably due to the enzyme having a drainage facilitating ability^23^. Conversely, if the enzyme was unable to react with the fiber, then only a slight increase in pulp freeness was observed. When the NaOH dosage was higher, along with increasing enzyme dosage, the before refining pulp freeness decreased from 520 to 421 mL, with a downward trend.
  Figure 5 shows the semi-chemical mechanical pulp group performances of the 2^3^ factorial rice straw designs after refining the pulp freeness values. Each item of variable is described in Table 2. The midpoint sets were subjected to 3 replications. The average post-refining freeness was 165 mL, with a standard deviation of 29.7 mL. Table 4 shows the factorial design of the effects of variables. At a 95% confidence level, the t value distribution was at 2.160 percentage points. The main effects and interactions were evaluated to be 18.0. After the factorial design was analyzed, we found that 3 items, i.e., main effect A (NaOH dosage), main effect C (refining time), and interaction AB (NaOH dosage and enzyme dosage), were significant. The probable causes were that an increase in refining time allowed rice straw fibers to be effectively crushed and fibrillated, further causing pulp freeness to decrease. Therefore, with an increase in refining time, pulp freeness had an apparent downward trend. When the NaOH dose increased, pulp freeness also showed an apparent downward trend, whereas an increase in enzyme dosage caused the pulp freeness to decrease less. This result indicated that reactions of NaOH with rice straw initiated degradation of the lignin structure and caused fibers to soften and swell, which enhanced the enzymatic reaction efficiency, facilitating rice straw fibers to proceed in refining and reducing the pulp freeness. These results illustrated the interactive effect of NaOH dosage and enzyme dosage. Of the experimental sets the post refining freeness of 602 mL for (−, −, +) = (5%, 0.2%, 2 h), This compared to (−, −, −) = (5%, 0.2%, 1 h) pulp with 588 mL freeness seemed odd, as under the same pretreatment conditions, along with the increase in refining time, the freeness increased instead. We made an extra effort to explore this and found that upon completion of refining, the pulp freeness of rice straw semi-chemical mechanical pulp increased with storage time. The use of 85°C hot water and readjustment of the disintegrator were unable to remove the latency and hence the phenomenon. We deemed that the higher freeness of the set (−, −, +) = (5%. 0.2%, 2 h) might be due to prolonged storage of the rice straw pulp.
3. Rate of acceptable fiber
  Figure 6 shows the 2^3^ factorial design experiments on the accepted fiber rates of the results. The post-refined rice straw semi-chemical mechanical pulps were screened, and the portions passing the 20 mesh screen and retained on the 100 mesh screen were the accepted fiber fraction. The individual variables are described in Table 2. The midpoint set would undergo 3 replications. The average accepted fiber rate was 32.3%, and the standard deviation was 0.5%. Table 5 shows the variable values of acceptable fiber fractions. At a confidence interval of 95%, each two repeat experiments gave a t value distribution percentile effective evaluation of 4.5. Through factorial analysis, the main effect A and interaction AB were the two items with significant effects. Therefore, when the NaOH dosage increased, the acceptable fiber fraction of the rice straw also increased. However, NaOH dosage and enzyme dosage interact with one another. We surmise that the enzymatic reaction efficiency was determined by whether a sufficient NaOH dosage had taken part in the pretreatment to initiate destruction of lignin structures and cause the fibers to become soft and swollen; then, the enzyme could gain entry into the fiber cell walls of the rice straw interior and to obtain better reaction efficiency.

### Handsheet properties

Rice straw after thermal alkali and enzymatic pretreatments was beaten with a Hollander to prepare the rice straw semi-chemical mechanical pulps. The results of the handsheet properties presented in the factorial design scheme are shown in Table 6. The central point condition (group 9) with 3 replications gave pulp tensile index standard deviation of 1.38 Nm/g, burst index standard deviation of 0.04 kPa*m^2^/g, and ring crush index standard deviation of 0.18 kgf*m^2^/g. Each set was a blend of rice straw pulp and OCC pulp in a 1:1 ratio, which was then compared with the handsheet properties of 100% OCC pulps.

1. Tensile index
  The effects of various rice straw semi-chemical mechanical pulp handsheets on their tensile indices are shown in Fig. 7. The midpoint sets were replicated 3 times. The average tensile index was 27.43 N*m/g, and the standard deviation was 1.32 N*m/g. According to the set results of 1 to 9, we can discern that when the NaOH dosage increased from 5 to 10%, the tensile index of the handsheet gained a significant increase. Among the sets, set 6 (10%, 0.2%, 2 h) gave the best tensile index, with a gain of 167%. Set 2 (10%, 0.2%, 1 h) was the next best. It appeared that enzyme dosage and increase in refining time had no significant effect on the tensile index of the handsheets. These results were largely congruent with the fiber morphologies observed. When the NaOH dosage was 10%, the fibrillation of the fiber was high and coarse, which were helpful to increase the tensile strength of the pulp. When handsheets blended with rice straw semi-chemical mechanical pulp were compared to 100% OCC handsheets, at 5% NaOH dosage, the tensile index of the handsheets was slightly inferior, approximately 81% to 96% of the pure OCC pulp. When the NaOH dosage was 10%, however, the tensile index of the handsheets increased significantly and was 143% to 167% of the OCC pulp.

**Figure 7:**
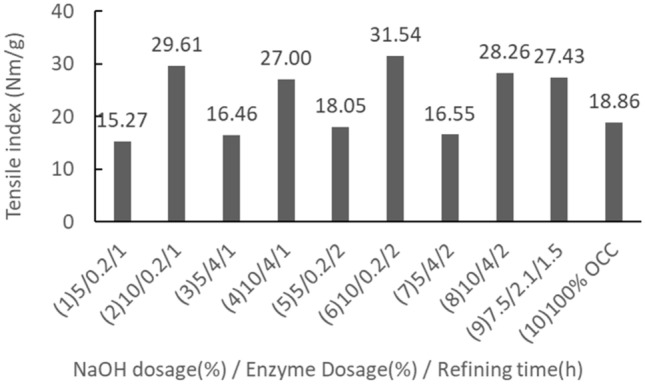
The effects of rice straw semi-chemical mechanical pulp experimental sets with respect to their handsheet tensile indices.
2. Burst index
  The effects of rice straw semi-chemical mechanical pulp experimental sets with respect to their burst indices are illustrated in Fig. 8. The midpoint set was replicated 3 times. The average paper burst index was 1.29 kPa*m^2^/g, and the standard deviation was 0.04 kPa*m^2^/g. Based on the results of Sets 1 to 9, we can discern that the burst index behaved roughly similarly to the tensile index. When the NaOH dosage was raised from 5 to 10%, the burst index of the handsheet showed a significant increase, particularly in Set 6 (10%, 0.2%, 2 h), which was the best. Set 2 (10%, 0.2%, 1 h) was the next best. However, the enzyme dosage and refinement time increases did not appear to exert apparent effects on the burst strength. Compared to blended rice straw semi-chemical mechanical pulp with the case of 100% OCC pulp, when the NaOH dosage was 5%, the burst index of the handsheet decreased slightly to the 77% and 89% ranges; however, when the NaOH dosage was 10%, the burst index showed a significant increase to the 130% and 162% ranges.

**Figure 8:**
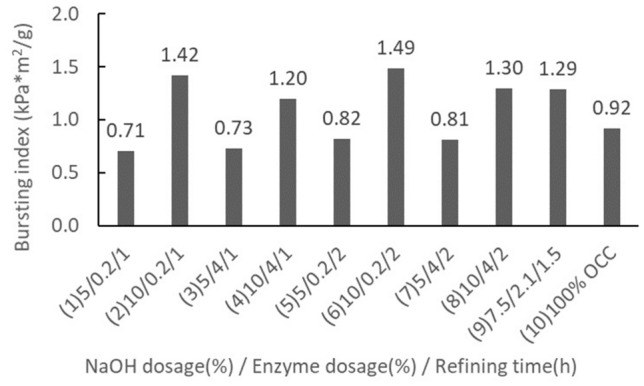
The effects of rice straw semi-chemical mechanical pulp on the burst index of handsheets.
3. Ring crush index
  The effects of rice straw semi-chemical mechanical pulp with respect to their handsheet ring crush index are shown in Fig. 9. The midpoint set underwent 3 replications. The average ring crush index was 8.11 kgf*m^2^/g, and the standard deviation was 0.18 kgf*m^2^/g. According to the results from sets 1 to 9, when the NaOH dosage increased from 5 to 10%, the ring crush strength was significantly enhanced, and set 7 (7.5%, 2.1%, 1.5 h) produced the best ring crush index, with a gain of 162%. Set 4 (10%, 0.2%, 1 h) was the next best. Increases in enzyme dosage and refinement time appeared to have no significant effects. Comparing the handsheets blended with rice straw semi-chemical mechanical pulp to 100% OCC handsheets, the ring crush index was apparently increased. When the NaOH dosage was 5%, the ring crush index of the rice straw/OCC handsheets showed no apparent change, roughly between −0.1% and 17%. When the NaOH dosage was 7.5%, however, the ring crush index of rice straw/OCC handsheets was at their best, with an average gain of 157%. If the NaOH dosage was further increased to 10%, the ring crush index of the handsheets increased 131% to 153%, showing a slight decreasing trend with a further increase in the NaOH dosage.

**Figure 9:**
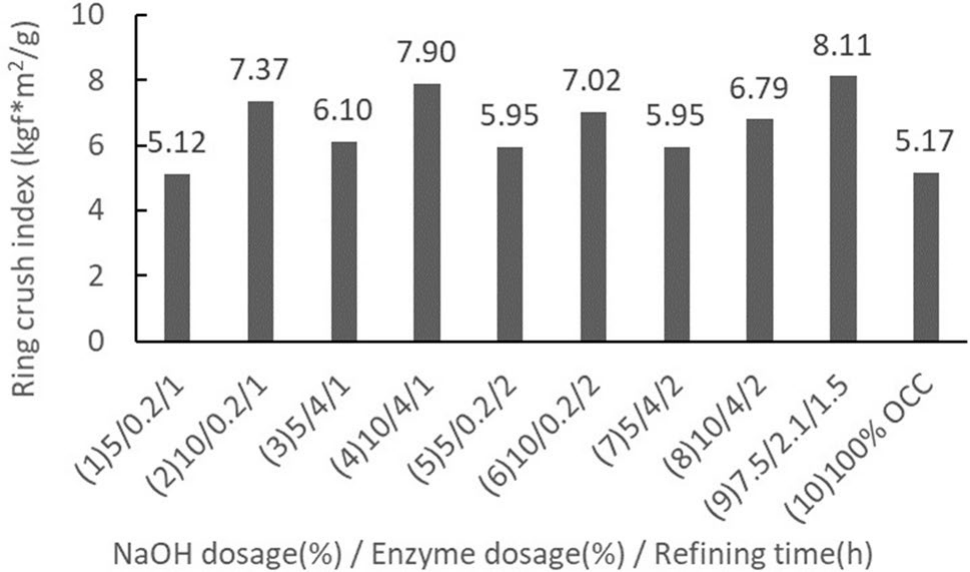
The effects of rice straw semi-chemical mechanical pulp on the ring crush index of handsheets.

**Table 7:** The handsheet physical properties after the best refining time conditions.

Group	Blending ratio (OCC/Rice straw)	NaOH dosage	Enzyme dosage	Refining time	Tensile index	Ring crush index	Burst index
		(%)	(%)	(times)	(N*m/g)	(kgf*m^2^/g)	(kPa*m^2^/g)
1	100/0	–	–	–	18.86	0.92	5.17
2	75/25	10	0.2	1	20.54	1.04	6.43
3	50/50				26.73	1.19	7.95
4	25/75				35.70	1.54	8.64
5	0/100				37.69	1.81	9.65
4. Evaluation of the optimal conditions
  From the results of factorial design analysis, when the NaOH dosage was 10%, the enzyme dosage and refinement time needed to increase relatively, and only at the lowest dosage of 0.2% and refinement time of 1 h could the objective of reducing rice straw pulp freeness and maintaining an acceptable fiber rate be achieved. The pulp freeness was 220 mL, and the acceptable fiber rate was 44.7%. The handsheet physical properties were also significantly increased. Compared to 100% OCC handsheets, the tensile index gained 157%, burst index gained 154%, and ring crush index gained 143%. Hence, we decided that Set 2 (10%, 0.2%, and 1 h) was the best pulping condition.

### The second stage: pilot scale test

In this stage, we used a pilot-scale 12″ single disc refiner to treat the best pretreated rice straw using a 005 knife plate for the task. The operational conditions of the single-disc refiner were to input 350 g rice straw with 10% NaOH at 60℃ and 5.8% consistency reacted for 2 h. After adjusting pH, 0.2% enzyme at 60℃and the same consistency was reacted with the rice straw for 2 h, finally, 4 passes of the pulp through the single-disc refiner to give the rice straw pulp. The accepted fiber rate was 36.5%, and the pulp freeness was 416 mL, which was different from the first-stage study of a target freeness of 220 mL.

**Figure 10:**
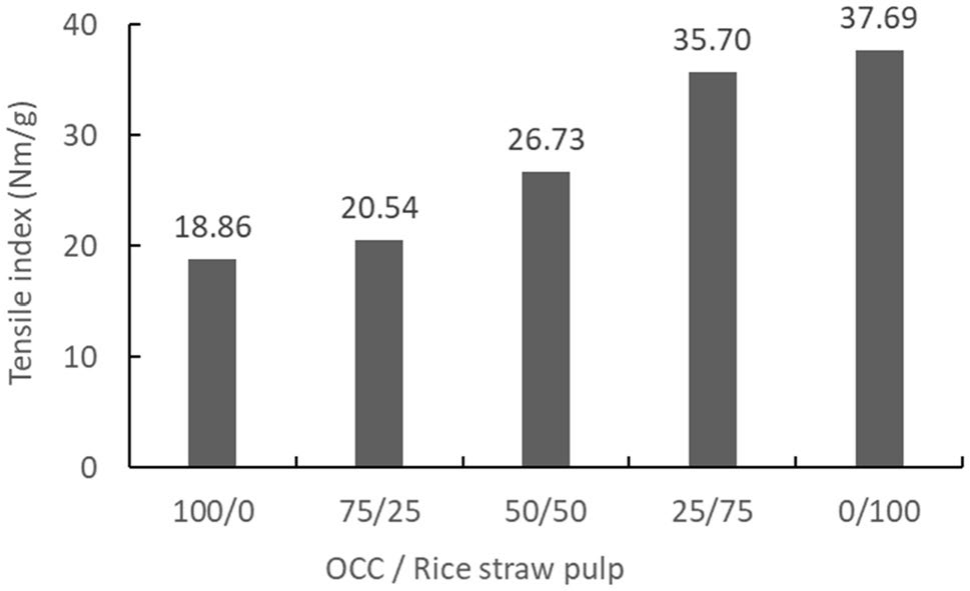
The effect of blending ratios of rice straw semi-chemical mechanical pulp with OCC on the tensile indices of the handsheets (expanded experiments).

**Figure 11:**
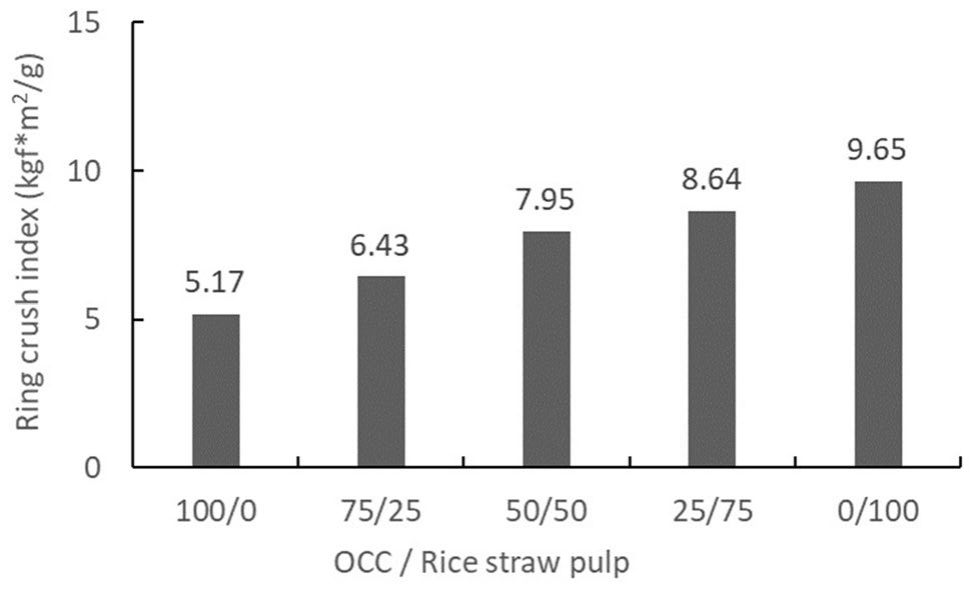
The effect of blending ratios of rice straw semi-chemical mechanical pulp with OCC on the burst indices of the handsheets (expanded experiments).

**Figure 12:**
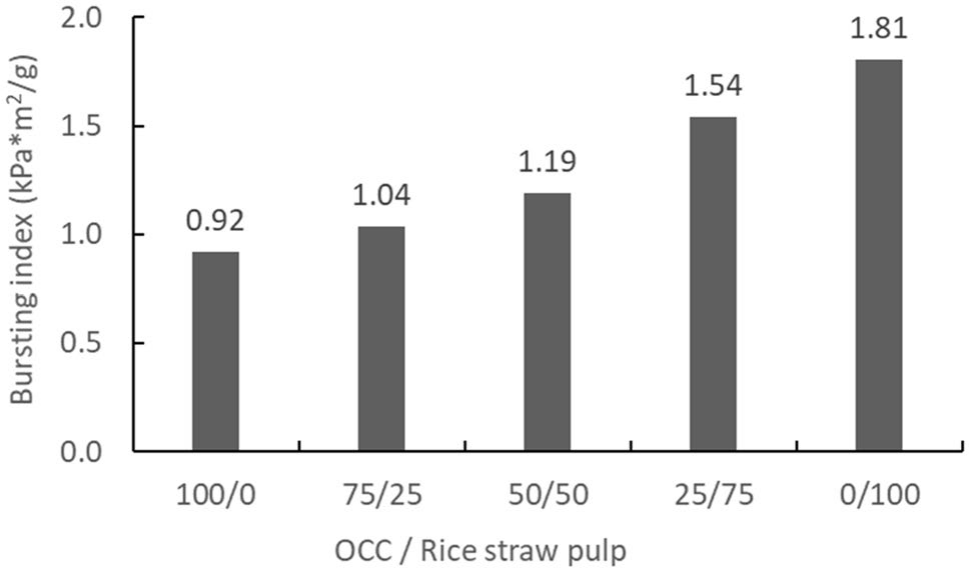
The effects of blend ratios of rice straw mech. pulp and OCC on the ring crush indices of the handsheets (expanded experiments).

1. Physical properties of the handsheets
  Blended rice straw semi-chemical mechanical pulp and OCC (0%, 25%, 50%, 75%, and 100%) were formed into handsheets of 100 gsm. Then, according to related property testing and formula calculations, the handsheet tensile, burst, and ring crush strengths were compared, and the results are shown in Table 7. Additionally, the rice straw semi-chemical mechanical pulp and OCC pulp blending ratio effects are shown in Figs. 10, 11, and 12. From these results, compared to 100% OCC pulp, along with an increase in rice straw pulp, the handsheet tensile, burst, and ring crush indices all exhibited linear increases. The tensile index increased 109–200%, the burst index increased 113% to 196%, and the ring crush index increased 124 to 187%. Hence, the handsheet physical properties showed huge increases as a result of blending.

### The third stage: Commercial paper molding company online tests

**Figure 13:**
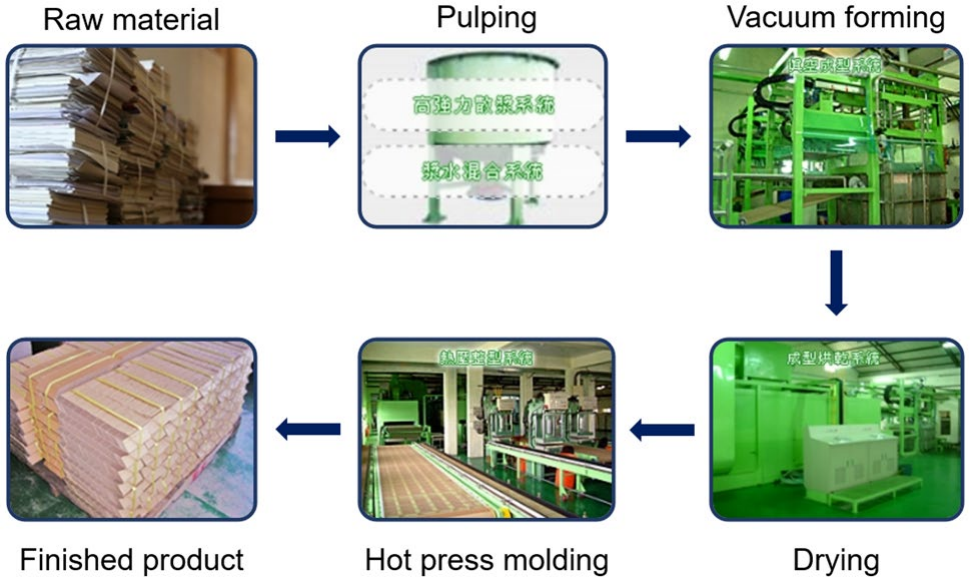
The production flow of Chang-Tai paper mold products.

**Figure 14:**
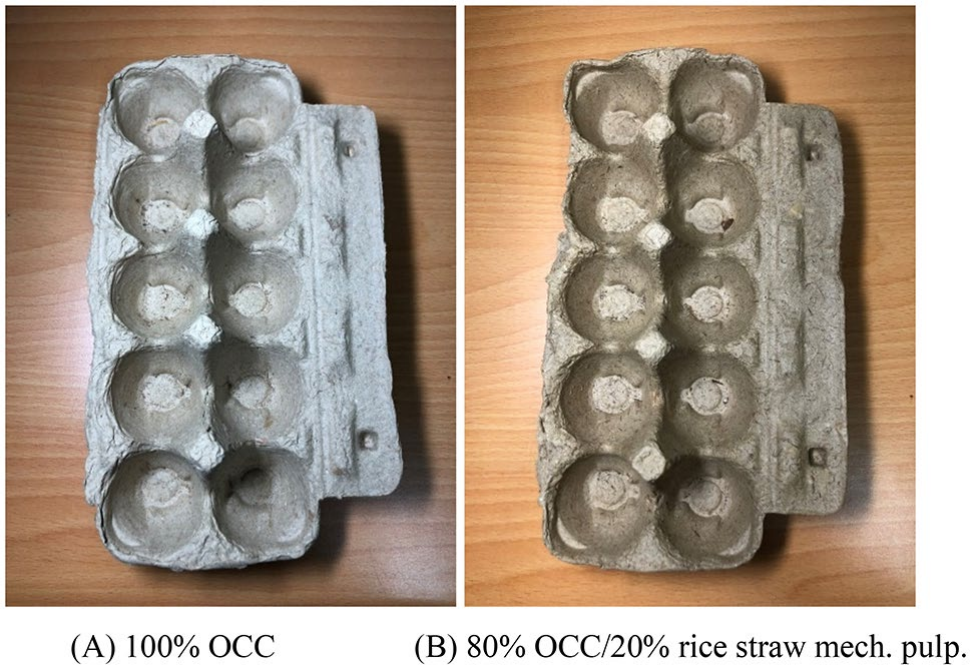
Egg carton paper molds produced by Chang-Tai Inc.

Sixty kg of rice straw pulp was delivered to the cooperating Chang-Tai Co. Upon blending with the OCC pulp of the mill, egg carton paper molding production proceeded. The production flow of Chang-Tai Co. is illustrated in Fig. 13. The raw materials after pulp disintegration were turned into pulp, and then vacuum was applied to the molding to form the products. After drying, hot press shaping was applied according to demand and to strengthen external shaping and structural strength to complete the paper molding production. The paper molding company produces 100% OCC-based and 20% rice straw pulp blended paper molding egg cartons, as shown in Fig. 14. Based on the photos, there was no apparent difference in the external smoothness of the 100% OCC-based product and the product blended with 20% rice straw pulp. Furthermore, according to the on-site testing report, when the rice straw pulp blending ratio was 20%, the operational parameters, such as pulp suction time, demolding time and drying time, were also not significantly different from the original 100% OCC-based production. When the addition ratio is greater than 20%, it will affect the appearance of the mold products. Therefore, the use of 20% rice straw semi-chemical mechanical pulp apparently exerted no noticeable effects on the on-site operation and external appearance of the products.

### Hypothetic mechanism

**Figure 15:**
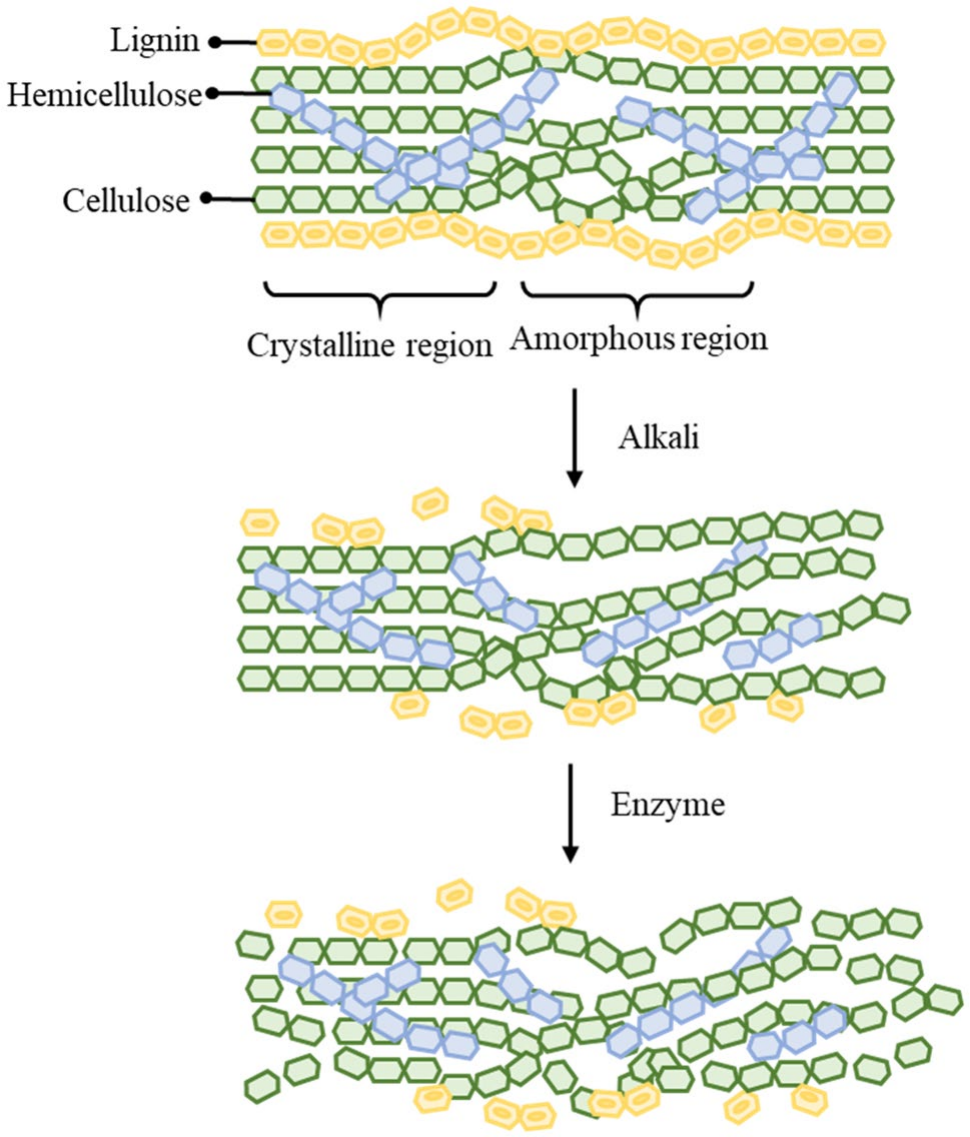
The hypothetical reaction mechanisms of alkali and enzyme with rice straw lignocellulose^16,17,21^.

Based on the experimental results of this study, the interaction AB (NaOH and enzyme dosages) had significant effects on the resulting rice straw pulp freeness and acceptable fiber rate. We propose a hypothetical mechanism to explain the performance, as shown in a schematic diagram in Fig. 15. When the NaOH dosage is suboptimal, the protective outer layer of lignin on the outside of rice straw is undamaged, causing the subsequent enzyme treatment to be unable to effectively enter the fibers to react. Leading to rice straw pulp of inferior quality. If, however, there is sufficient NaOH to pretreat rice straw and degrade the exterior of cellulosic bundles, then the enzymatic reaction efficiency with cellulose can be enhanced, resulting in superior rice straw semi-chemical mechanical pulp properties^16,17,21^.

## Conclusion

This study separately used a laboratory Hollander beater, expanded to a pilot-scale 12” single-disc refiner, and finally a commercial paper mold mill online test to investigate the feasibility of using thermal-alkali/enzymatic pretreated rice straw semi-chemical mechanical pulp to substitute for old corrugated carton (OCC). When the NaOH dosage, enzyme dosage, and refinement time increased, the rice straw pulp showed a significant decrease in pulp freeness and an increase in the acceptable fiber rate. The enzyme dosage must work under the premise of sufficient NaOH dosage to begin with, then it reacted with rice straw pulp to enhance rice straw quality. Finally, a pilot-scale single-disc refiner was simulated to replace the Hollander beater and produce the best manufacturing condition for rice straw semi-chemical mechanical pulp. Along with the increase in the blending ratio of rice straw semi-chemical mechanical pulp, the handsheet tensile index increased 109% to 200%, the burst index increased 113% to 196%, and the ring crush index increased 124% to 187%. The paper strengths gained vast increases. When blended rice straw pulp and OCC pulp were formed into egg carton molds, compared to the original OCC pulp, there was no apparent on-site operational or product appearance problem. These results amply illustrated that thermally alkali/enzymatically pretreated rice straw semi-chemical mechanical pulp has the potential to substitute for OCC pulp.

## Data Availability

The datasets presented in this current study are available from the corresponding author on reasonable request.
